# A Novel Just-in-Time Learning Strategy for Soft Sensing with Improved Similarity Measure Based on Mutual Information and PLS

**DOI:** 10.3390/s20133804

**Published:** 2020-07-07

**Authors:** Yueli Song, Minglun Ren

**Affiliations:** 1 School of Management, Hefei University of Technology, Hefei 230009, China; song052414@mail.hfut.edu.cn; 2 Key Laboratory of Process Optimization and Intelligent Decision-making, Ministry of Education, Hefei 230009, China

**Keywords:** just-in-time learning, locally weighted partial least squares, mutual information, similarity measure, soft sensor

## Abstract

In modern industrial process control, just-in-time learning (JITL)-based soft sensors have been widely applied. An accurate similarity measure is crucial in JITL-based soft sensor modeling since it is not only the basis for selecting the nearest neighbor samples but also determines sample weights. In recent years, JITL similarity measure methods have been greatly enriched, including methods based on Euclidean distance, weighted Euclidean distance, correlation, etc. However, due to the different influence of input variables on output, the complex nonlinear relationship between input and output, the collinearity between input variables, and other complex factors, the above similarity measure methods may become inaccurate. In this paper, a new similarity measure method is proposed by combining mutual information (MI) and partial least squares (PLS). A two-stage calculation framework, including a training stage and a prediction stage, was designed in this study to reduce the online computational burden. In the prediction stage, to establish the local model, an improved locally weighted PLS (LWPLS) with variables and samples double-weighted was adopted. The above operations constitute a novel JITL modeling strategy, which is named MI-PLS-LWPLS. By comparison with other related JITL methods, the effectiveness of the MI-PLS-LWPLS method was verified through case studies on both a synthetic Friedman dataset and a real industrial dataset.

## 1. Introduction

Data-driven soft sensors are usually models built based on a large quantity of data generated in production processes and can be used to monitor key indicators that are difficult to measure [1,2]. With the development of big data and other information technologies, data acquisition and processing in industrial processes have become easier, which makes data-driven soft sensors very popular in industrial process monitoring, quality prediction, and other process control-related tasks [3,4,5].

Traditional soft sensor models usually adopt some linear or non-linear methods, including partial least squares [6,7], support vector machine [8,9], artificial neural network [10,11], etc. These are basic kinds of global modeling methods since the models are built offline based on historical data. A characteristic feature of a global model is that once the model is established, it is difficult to adaptively adjust to a change of processes, so its performance may gradually degrade in practical application. To solve this issue, adaptive methods such as moving window [12,13,14], time difference [15,16], and recursive methods [17] are proposed. Although these methods can adapt to slow changes in processes to some extent, they cannot deal with abrupt changes [18,19].

Just-in-time learning (JITL) provides a new way to deal with the changing characteristics of processes. Different from the global methods, the JITL-based method does not build models in advance but only stores the historical sample data. When the prediction is needed, the JITL-based method performs the following three steps [19,20,21]: first, the nearest neighbor samples for a query point based on the similarity measure are selected; second, the selected nearest neighbor samples are used to establish a local model; third, the predicted value according to the model is calculated, and then the model is discarded; and the above three steps are repeated when the next query comes. It can be seen that the selection of the nearest neighbor samples and the establishment of the local model are two important steps. Only by selecting the sample points that are close to the query point can we establish an effective local model and obtain high prediction accuracy. The selection of sample points depends on the similarity index, so the similarity between samples should be defined carefully.

At present, there are many methods to measure the similarity between samples in JITL. Euclidean distance (ED) [22], weighted Euclidean distance (WED) [23,24,25], and distance and angle methods are some usual methods [21]. Correlation-based similarity measurement methods were also proposed by Japanese scholars in recent years [26,27]. These methods have been proved to be effective in some applications. However, the output information of samples is ignored completely in a similarity measure, which makes these methods inaccurate and causes a larger deviation in the nearest neighbor sample selection. By using a global model to estimate the query output first, the output information of historical data and query point can both be used to define the similarity [28,29]. However, the accuracy of the global model used in this method is usually not high, and the estimation error also has a negative impact on the selection of nearest neighbor samples. In order to make full use of information of samples, and avoid the introduction of additional estimation errors, Yuan [19] proposes a supervised latent structure method, which uses partial least squares (PLS) to project the historical samples and the query point into a low-dimensional latent variable space and uses Euclidean distance to calculate the similarity in the latent variable space. However, this method relies on the PLS model, which cannot deal with nonlinearity or non-Gaussian distribution. Considering different effects of input variables on output, a WED method based on mutual information (MI) has been proposed in the literature [30,31]. MI can not only capture the linear or nonlinear correlation between variables but is also not limited by the data distribution characteristics, so the above MI-based WED method gives a more accurate similarity measure than traditional methods. However, when input variables are multicollinear, the MI-based method may not be satisfactory. Especially in the case of high-dimensional input, collinearity will not only make the calculation of the whole model complex but also affect the accuracy of the similarity measure.

Through the above literature review, it can be seen that the different contributions of input variables to output, the correlation between input variables, the redundancy of input variables, and other factors can affect the similarity measure between samples. Especially in the case of high-dimensional input, the collinearity or redundancy between input variables can also reduce the efficiency of similarity calculation. To overcome the shortcomings of the above methods in the similarity measure, a novel MI-PLS-based similarity measure is proposed in this paper by skillfully combining MI and PLS. By calculating mutual information between input and output variables and realizing variable weighted based on MI, the correlation between input and output variables can be accurately described, and some uncorrelated redundant variables can be removed at the same time. Then, by using PLS to project the weighted variables into a low dimensional space, we can eliminate the influence of collinearity between variables on the similarity measure, and the computational efficiency can also be improved by reducing the dimension. In order to apply the proposed similarity measure method to soft sensor modeling in the JITL framework, a two-stage strategy was also designed to improve the computational efficiency of online prediction by avoiding repeated calculation as much as possible. The main calculation steps are as follows: in the training stage, MI between input and output variables is firstly calculated, then each variable is weighted based on its MI value. After that, the irrelevant variables with zero weights are removed. Finally, PLS is used to project the remaining weighted variables into latent variable space. In the prediction stage, for a query point, the similarity and sample weights are calculated by the ED-based method in the latent variable space. Then, locally weighted partial least squares (LWPLS), with variables and samples double-weighted, are used for local modeling and answering a prediction query. All the above calculation processes constitute a novel JITL soft sensor modeling strategy, which is named MI-PLS-LWPLS. By using MI, correlation information between input and output is described accurately in a similarity calculation. At the same time, PLS is used to overcome the influence of collinearity, and the double weighting method is used to describe the different importance of variables to output and historical samples to query a sample in local modeling [19]. Therefore, the proposed method can achieve high prediction accuracy. The effectiveness of MI-PLS-LWPLS was verified by using both numerical and industrial cases. The proposed similarity measure method is generally applicable to soft sensor modeling in the JITL framework. Although LWPLS was chosen to build the local model in this study, when the process has strong non-linear characteristics, it may be necessary to select methods such as Gaussian process regression (GPR) or support vector regression (SVR) to build a local model. At this time, the proposed similarity measure method can still be considered in combination with these methods by selecting accurate neighbor samples to achieve a prediction model with good performance.

This paper is arranged as follows: Section 2 briefly reviews MI and LWPLS. Section 3 introduces the proposed method in detail. Section 4 verifies the effectiveness of the proposed method through numerical and industrial cases. Conclusions are made in Section 5.

## 2. Preliminaries

### 2.1. Mutual Information

MI between two random variables can represent the degree of their interdependence. The larger the MI value is, the more relevant the two variables are. Compared with traditional correlation criteria, such as correlation coefficient, cross-correlogram, etc., MI can describe the correlation among variables more comprehensively, including linear, periodic, or nonlinear correlation [32].

Given two random variables X and Y, the MI between them is defined as follows [33]:

(1) $$I(X;Y)=\iint \mu (x,y)\log\frac{\mu (x,y)}{\mu_{X}(x)\mu_{Y}(y)}dxdy$$

Here, $\mu_{X}(x)$ and $\mu_{Y}(y)$ are marginal probability distributions, and μ(x,y) is the joint probability distribution. To calculate MI, the probability density functions (PDFs) need to be estimated first. The commonly used methods to calculate MI based on PDF estimation are histogram and kernel-based estimators. However, it is not easy to estimate the PDFs of random variables accurately in practical applications. Kraskov et al. [33] proposed a K-nearest neighbor (K-NN) method to directly calculate MI from data samples and avoid PDF estimation. This greatly reduces the complexity of the MI calculation.

Consider a new space *Z* = (*X, Y*), which is built from the original variables *X* and *Y*; for any point *z_i_* = (*x_i_*, *y_i_*), *I* = 1, 2, …, *N*, the distance from point *z_i_* to its K-nearest neighbor *z_k_* = (*x_k_*, *y_k_*) is defined as follows:

(2) $$D_{k}=\frac{\varepsilon (i)}{2}=\max(\left\|x_{i}-x_{k}\right\|,\left\|y_{i}-y_{k}\right\|)$$

Then, for other points, *z_j_* = (*x_j_*, *y_j_*), (*j* ≠ *i*) in Z space, count *n_x_*(*i*) the number of the points *x_j_* that satisfy $\left\|x_{i}-x_{j}\right\|\leq \varepsilon (i)/2$ and *n_y_*(*i*) the number of the points *y_j_* that satisfy $\left\|y_{i}-y_{j}\right\|\leq \varepsilon (i)/2$. Then, MI can be calculated by the following formula:

(3) $$I(X;Y)=\psi (K)-\frac{1}{N}\sum_{i=1}^{N}\left(\psi (n_{x}(i)+1)+\psi (n_{y}(i)+1)\right)+\psi (N)$$

where *ψ*(x) is the digamma function satisfying *ψ*(x + 1) = *ψ*(x) + 1/x, *ψ*(1) = −C, C = 0.5772156…. Parameter K denotes the number of neighbors and is usually set to be an integer in the range of 2 to 8 [33,34].

### 2.2. Locally Weighted PLS

LWPLS, which combines PLS with local learning, can deal with nonlinearity and collinearity and is a very commonly used JITL modeling method [35,36].

For the input and output variables $X\in {\mathbb{R}}^{N\times p}$ and $Y\in {\mathbb{R}}^{N\times 1}$, *N* is the sample number, and *p* represents the input variable number. The *n*th sample is denoted by (***x**_n_*,y*_n_*), $y_{n}\in \mathbb{R}$, $x_{n}\in {\mathbb{R}}^{1\times p}$ defined by:

(4) $$x_{n}=[x_{n1},x_{n2},\cdots ,x_{np}]$$

To estimate the output of a new sample $x_{q}\in {\mathbb{R}}^{1\times p}$, firstly, *d_n_* representing ED between ***x**_q_* and ***x**_n_* is calculated. Based on this, the sample weight or sample similarity *s_n_* is defined as follows:

(5) $$d_{n}=\sqrt{(x_{n}-x_{q}){\left(x_{n}-x_{q}\right)}^{T}}$$

(6) $$s_{n}=\exp(-\frac{d_{n}}{h\times \sigma_{d}})$$

where *σ_d_* is the standard deviation of $D=[d_{1},d_{2},\cdots ,d_{N}]$, and *h* is called bandwidth, which can control the speed of weight attenuation. The smaller *h* is, the faster the weight decays; on the contrary, the larger *h* is, the slower the weight decays [35,36,37]. Then, an *N* × *N* matrix **Ω** is built as follows:

(7) $$\Omega =diag[s_{1},s_{2},\ldots ,s_{N}]$$

Generally, the output estimation of ***x**_q_* is obtained by the following calculation steps 1–11 [37,38]:

1: Set the number of latent variables *R* and the tuning parameter *h*;
2: Calculate **Ω**;
3: Calculate ***X***_0_, ***Y***_0_, and ***x**_q,0_*;
  (8) $$X_{0}=X-1_{N}[{\bar{x}}_{1},{\bar{x}}_{2},\cdots ,{\bar{x}}_{p}]$$
  (9) $$Y_{0}=Y-1_{N\times 1}\bar{y}$$
  (10) $$x_{q,0}=x_{q}-[{\bar{x}}_{1},{\bar{x}}_{2},\cdots ,{\bar{x}}_{p}]$$
  (11) $${\bar{x}}_{i}=\frac{\sum_{n=1}^{N}s_{n}x_{ni}}{\sum_{n=1}^{N}s_{n}}$$
  (12) $$\bar{y}=\frac{\sum_{n=1}^{N}s_{n}y_{n}}{\sum_{n=1}^{N}s_{n}}$$
4: Initialize: ***X**_r_* = ***X***_0_, ***Y**_r_* = ***Y***_0_, ***x**_q,r_* = ***x**_q,_*_0_, ${\overset{⌢}{y}}_{q}=\bar{y}$;
5: For *r* = 1: *R*;
6: Calculate the weight loading ***W**_r_*;
  (13) $$W_{r}=\frac{X_{r}^{T}\Omega Y_{r}}{\left\|X_{r}^{T}\Omega Y_{r}\right\|}$$
  Derive the *r*th latent variables.
  (14) $$t_{r}=X_{r}W_{r},t_{q,r}=x_{q,r}W_{r}$$
7: Derive *X*-loading vector ***p***_r_ and *Y*-regression coefficient *q*_r_;
  (15) $$p_{r}=X_{r}^{T}\Omega t_{r}/t_{r}^{T}\Omega t_{r},q_{r}=Y_{r}^{T}\Omega t_{r}/t_{r}^{T}\Omega t_{r}$$
8: Update ${\overset{⌢}{y}}_{q}={\overset{⌢}{y}}_{q}+t_{q,r}q_{r}$;
9: Update ***X**_r_*_+1_, ***Y**_r_*_+1_, and ***x**_q,r_*_+1_;
  (16) $$X_{r+1}=X_{r}-t_{r}p_{r}^{T}$$
  (17) $$Y_{r+1}=Y_{r}-t_{r}q_{r}$$
  (18) $$x_{q,r+1}=x_{q,r}-t_{q,r}p_{r}^{T}$$
10: End for;
11: Output ${\overset{⌢}{y}}_{q}$.

## 3. The Proposed Method

Firstly, the similarity measure based on PLS latent structure proposed by Yuan [19] is briefly described. On this basis, the proposed JITL method with the MI-PLS-based similarity measure is introduced in detail.

### 3.1. PLS-Based Similarity Measure

The PLS-based similarity measure method calculates the similarity by using ED in latent variable space. Suppose that $X\in {\mathbb{R}}^{N\times p}$ and $Y\in {\mathbb{R}}^{N\times 1}$ are input and output variables. The calculation formulas of the PLS algorithm are defined as:

(19) $$\begin{array}{l} X=T^{T}P+E\\ Y=U^{T}Q+F \end{array}$$

$T\in {\mathbb{R}}^{N\times R}(1\leq R\leq p)$ represents the latent variable score matrix of input space. Let $T_{j}\in {\mathbb{R}}^{N\times 1}$ (*j* = 1, 2, …, *R)* represent the *j*th latent variable and $t_{n}\in {\mathbb{R}}^{1\times R}$ (*n* = 1, 2, …, *N*) represent the *n*th sample in latent variable space, i.e., $T=[T_{1},T_{2},\ldots ,T_{R}]={\left[t_{1},t_{2},\ldots ,t_{N}\right]}^{T}$, and ***t**_q_* represents the query sample. Then, the ED between ***t**_q_* and ***t**_n_* can be calculated as follows:

(20) $$d_{n,LV}=\sqrt{(t_{n}-t_{q}){\left(t_{n}-t_{q}\right)}^{T}}$$

On this basis, the weight of the *n*th sample is defined as:

(21) $$S_{n,LV}=\exp(\frac{-d_{n,LV}}{h\sigma_{d}})$$

Here, *h* is the tuning parameter, which is also known as bandwidth. $\sigma_{d}$ is the standard deviation of $d_{n,LV}$ (*n* = 1, 2, …, *N*).

### 3.2. The Proposed MI-PLS-LWPLS Method

Compared with traditional similarity measure methods, which only use input information, the PLS-based similarity measure introduced in Section 3.1 can select nearest neighbor samples more accurately by using supervised latent structure. However, PLS cannot describe a nonlinear correlation between input and output. Instead, mutual information can express both linear and nonlinear correlation at the same time, and it cannot be affected by data distribution. References [30,31] adopted an MI-based similarity measure, and the results showed that this method can obtain better prediction accuracy than the traditional similarity measure. However, MI cannot deal well with the redundancy caused by the correlation between input variables [39]. Therefore, in the case of multiple collinearities between input variables, the prediction results are not ideal.

**Figure 1:**
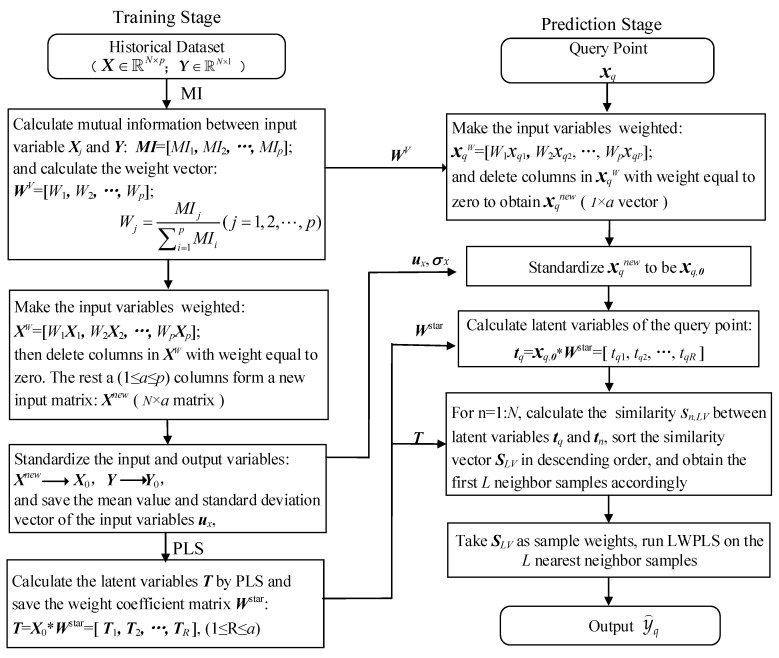
Two-stage flow chart of the proposed method.

To develop a JITL-based soft sensor with good performance in the case of nonlinearity and collinearity, a novel similarity measure method combining MI and PLS is proposed. In order to fully consider the different importance of variables to the output and samples to the query sample, a two-stage strategy was designed to realize double weighting variables and samples in building an LWPLS-based local model. Figure 1 gives the two-stage flow chart of the proposed method, which is termed MI-PLS-LWPLS.

#### 3.2.1. Training Stage

In the training stage, some important variables and parameters, such as MI between input variables and output, latent variables, and weight matrix are obtained by offline computing based on the historical dataset so as to prepare for online prediction. Detailed computing steps are given below.

Step 1: Calculate MI between each input variable *X_j_* and the output *Y* to obtain a mutual information vector *MI* = [*MI*_1_, *MI*_2_,…, *MI_p_*]. Then calculate the variable weight vector *W^V^* = [*W*_1_, *W*_2_,…, *W_p_*], here *W_j_* (*j* = 1, 2, …, *p*), as follows:

(22) $$W_{j}=\frac{MI_{j}}{\sum_{i=1}^{p}MI_{i}}$$

Step 2: Weight the input variables by using the weight vector ***W**^V^* and record the weighted input matrix as ***X**^W^*= [*W*_1 × 1_, *W*_2 × *2*,_ …, *W_p_**X**_p_*]. Then, remove all zero data columns caused by zero weight in ***X**^W^*, and form a new input matrix $X^{new}\in {\mathbb{R}}^{N\times a}$ with the remaining *a* columns.

Step 3: Standardize input matrix ***X**^new^* and output variable ***Y***, then record them as ***X***_0_ and ***Y***_0_, respectively. The formula is as follows:

(23) $$\begin{array}{l} x_{0,nj}=\frac{x_{nj}-u_{x,j}}{\sigma_{x,j}}\\ y_{0,n}=\frac{y_{n}-u_{y}}{\sigma_{y}} \end{array}$$

where *x_nj_* represents the element of row *n*, column *j* of input matrix ***X**^new^*. *u_x_*_,*j*_ and $\sigma_{x,j}$ respectively represent the element of column *j* of mean vector ***u***_x_ and standard deviation vector $\sigma_{x}$ of input matrix ***X**^new^*. $x_{0,nj}$ is the value of the standardized *x_nj_*. *u*_y_ and $\sigma_{y}$ are the mean and standard deviation of output ***Y***, respectively. The *n*th element *y_n_* of output ***Y*** is expressed as *y_0_,_n_* after standardization.

Step 4: Take ***X***_0_ and ***Y***_0_ as input and output variables, respectively. Then, the input latent variable matrix ***T*** is obtained by running PLS, and save the transformation weight coefficient matrix ***W**^star^*, where $T=X_{0}*W^{star}=[T_{1},T_{2},\ldots ,T_{R}]$, 1 ≤ *R* ≤ a.

#### 3.2.2. Prediction Phase

In the prediction stage, a query is responded to by the following calculation procedure. Firstly, parameters obtained in the training stage are used to complete the transformation calculation of the query sample, then a locally weighted model is established by using selected nearest neighbor samples to obtain predicted output value. Detailed computing steps are given below.

Step 1: Transform the query sample ***x**_q_* = [*x_q_*_1_, *x_q2_*, …, *x_qp_*] ($x_{q}\in {\mathbb{R}}^{1\times p}$) into ***x**_q_^W^*= [*W*_1_*x_q_*_1_, *W*_2_*x_q2_*, …, *W_p_x_qp_*] by using the weight vector ***W**^V^* obtained in step 1 of the training stage. Then, remove zero data columns caused by zero weight in ***x**_q_^W^*and record the processed query sample vector as ***x**_q_^new^*(${x_{q}}^{new}\in {\mathbb{R}}^{1\times a}$).

Step 2: Standardize query sample ***x**_q_^new^* to ***x**_q,_*_0_ according to the following equation:

(24) $$x_{q,0}=\frac{x_{q}-u_{x}}{\sigma_{x}}$$

Step 3: Project the query ***x**_q,_*_0_ into latent variable space to obtain ***t**_q_* by using the transformation weight coefficient matrix ***W***^star^ obtained in step 4 of the training stage. *R* is the latent variable number, 1 ≤ *R* ≤ *a*.

(25) $$t_{q}=x_{q,0}*W^{star}=[t_{q1},t_{q2},\ldots ,t_{qR}]$$

Step 4: In the latent variable space, calculate Euclidean distance between the query sample ***t**_q_* (1 × *R* vector) and each training sample ***t**_n_*(*n* = 1, 2, …, *N)*. Sample weights are also obtained according to the ED. Taking the *n*th sample as an example, the Euclidean distance *d_n,LV_* and the similarity *s_n,__LV_* between the *n*th sample and the query sample are calculated by Equations (20) and (21).

Step 5: Sort the similarity vector ***S**_LV_* in descending order and save the order index vector recorded as ***Ind***, and sort the training input matrix ***X**^new^* (*N* × *a*) obtained in step 2 of the training stage according to the ***Ind***. Then the first *L* samples in ***X**^new^* with the largest similarity value are selected as the nearest neighbor samples. Finally, an LWPLS-based model with a sample weighted by ***S**_LV_* is established, and the predicted output ${\overset{⌢}{y}}_{q}$ is then obtained by taking ***x**_q_^new^* as the query input.

It can be seen that in the above calculation process, the input variables in LWPLS are weighted by MI, and the nearest neighbor samples used for local modeling are also weighted by their similarity indexes. By performing double weighting operations, both variable importance and sample importance are considered [19]. Therefore, the proposed modeling method can accurately describe the complex relationship between input and output variables and achieve high accuracy.

## 4. Case Studies

In this section, the effectiveness of the proposed MI-PLS-LWPLS modeling method is verified through a numerical case on a Friedman dataset [40,41] and an industrial debutanizer column process (DCP) case. Three other LWPLS methods based on different similarity measures are used to compare with MI-PLS-LWPLS. The four modeling methods are as follows:

ED-LWPLS: Traditional Euclidean distance-based LWPLS (calculating sample similarity and weight in original input space).

PLS-LWPLS: PLS latent structure-based LWPLS (calculating sample similarity and weight in latent variable space).

MI-LWPLS: MI weighted Euclidean distance-based LWPLS (calculating sample similarity by using MI weighted ED in original input space and assigning sample weight accordingly).

MI-PLS-LWPLS: The proposed MI-PLS-based LWPLS (combining MI and PLS together in the similarity measure and weight assignment).

The prediction accuracy is measured by the criteria mean absolute relative error (MARE) and root mean square error (RMSE), defined as follows:

(26) $$\mathrm{MARE}=\frac{1}{M}\sum_{m=1}^{M}\left|\frac{y_{m}-{\hat{y}}_{m}}{y_{m}}\right|\times 100\%$$

(27) $$\mathrm{RMSE}=\sqrt{\frac{1}{M}\sum_{m=1}^{M}{\left(y_{m}-{\hat{y}}_{m}\right)}^{2}}$$

Here, $y_{m}$ and ${\overset{⌢}{y}}_{m}$ respectively represent the real and predicted values of the *m*th test point, and *M* represents the total sample number of the test dataset.

### 4.1. Numerical Experiment on Friedman Dataset

#### 4.1.1. Experimental Design

The Friedman dataset is defined by the equation below [40,41]:

(28) $$Y=10\sin(\pi X_{1}X_{2})+20{\left(X_{3}-0.5\right)}^{2}+10X_{4}+5X_{5}+\varepsilon$$

Here, *X*_1_ ~ *X*_10_ are random variables uniformly distributed in the interval [0,1], and *ε* is white noise of standard normal distribution. One can see that the output *Y* is related to the input variables *X*_1_ ~ *X*_5_, but not to *X*_6_ ~ *X*_10_.

The two cases below were investigated.

Case 1: Generate Friedman data based on the above Equation (28) and take *X*_1_ ~ *X*_10_ as the input and *Y* as the output to form a dataset.

Case 2: On the basis of case 1, add two input variables *X*_11_ and *X*_12_, which are determined by *X*_1_, *X*_2_, and *X*_3_ as follows:

(29) $$\begin{array}{l} X_{11}=0.5(X_{1}+X_{2})\\ X_{12}=0.5X_{3} \end{array}$$

We took *X*_1_ ~ *X*_12_ as the input and *y* as the output to form a new dataset. One can observe that in case 2, there are not only uncorrelated input variables *X*_6_~*X*_10_ but also redundant variables *X*_11_ and *X*_12_, which are collinear with *X*_1_, *X*_2_, and *X*_3_.

For the above two cases, 400 data samples were randomly generated, 300 of which were taken as training data and the remaining 100 as test data. The four modeling methods mentioned above were used in the experiment. The following parameters needed to be determined in the application of the four methods:

*L*: Number of neighbor samples used for local modeling in LWPLS;
*R*: Number of latent variables in LWPLS;
*h*: Tuning parameter in sample weight calculation;
*K*: Number of nearest neighbor samples used in *K*-NN-based MI estimation. *K* is usually an integer in the range of 2 to 8 [33,34].

**Figure 2:**
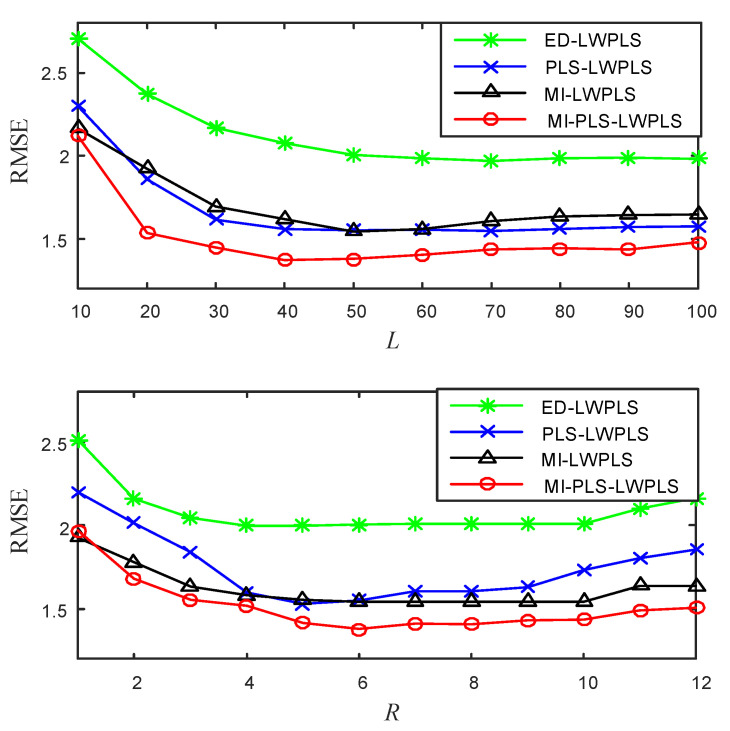
Influence of the changes of *L* and *R* on root mean square errors (RMSEs) of the four methods.

To determine parameters *L* and *R*, the influence of the changes of *L* and *R* on RMSE was studied by the cross-validation method. Figure 2 shows the results. The value of *L* varies from 10 to 100 with a step size of 10, and the value of *R* is an integer between 1 and 12.

From Figure 2, one can see that when the value of *L* is in the interval 40–100, the RMSEs of the four methods change very little. When *L* = 50, the four methods can obtain their own smaller RMSEs, so the *L* values of the four methods are all set to 50. The changes of RMSEs with *R* are similar to the above situation. When *R* changes between interval 5–10, the value of RMSEs fluctuates very little. When *R* is greater than 10, RMSEs tend to increase, indicating that the prediction results become worse. Therefore, *R* was set to six in this study according to the result shown in Figure 2.

Bandwidth parameter *h* was selected by trial-and-error experiments. Firstly, the initial value set of *h* was set as {0.01, 0.05, 0.1, 0.3, 0.6, 0.8, 1, 1.3, 1.6, 2, 5, 10, 20, 30, 50}. By minimizing the RMSE of the cross-validation experiment, an initial optimal *h* value could be obtained. Then, by further narrowing the selection range, a new value was set around the initial optimal *h* value, and finally, the optimal bandwidth parameter *h* was obtained by constantly narrowing the selection range and step size.

For parameter *K*, in order to avoid the inaccuracy in MI estimation caused by taking a specific *K* value, the following strategy was adopted: the mutual information with *K* set to be each integer in the interval 2–8 was calculated at first, and then the average of all MI values was taken as the final MI value.

#### 4.1.2. Results and Discussion

Table 1 gives statistical analysis results of prediction errors of the four modeling methods. It is observed that the MI-PLS-LWPLS method achieves minimum RMSE and MARE in both cases, which means that it has the best prediction performance. By further observing the results of the first method ED-LWPLS and the third method MI-LWPLS, their RMSE and MARE values in case 2 are both greater than those in case 1, which means the performance of these two methods in case 2 is worse than in case 1, while PLS-LWPLS and MI-PLS-LWPLS both perform better in case 2 than in case 1.

This is because two other collinear inputs *X*_11_ and *X*_12_ related to input *X*_1_, *X*_2_, and *X*_3_ are added in case 2. ED-LWPLS and MI-LWPLS select samples and define weights based on ED and MI weighted ED, respectively, both in the original sample space. These two methods cannot deal with collinear redundancy of input variables in the sample selection procedure, so their performance gets worse in case 2 than in case 1. PLS-LWPLS and MI-PLS-LWPLS calculate the sample similarity and weights in latent variable space based on PLS transformation, which can overcome the influence of collinearity, so they are more effective in case 2. In addition, the proposed method considers the different correlations between input and output variables in similarity calculation by weighting input variables based on MI, so more accurate neighbor samples are selected. In the local modeling phase, the variable and sample double-weighted modeling scheme is adopted. Therefore, MI-PLS-LWPLS achieves the best performance among the four methods in both cases.

**Figure 3:**
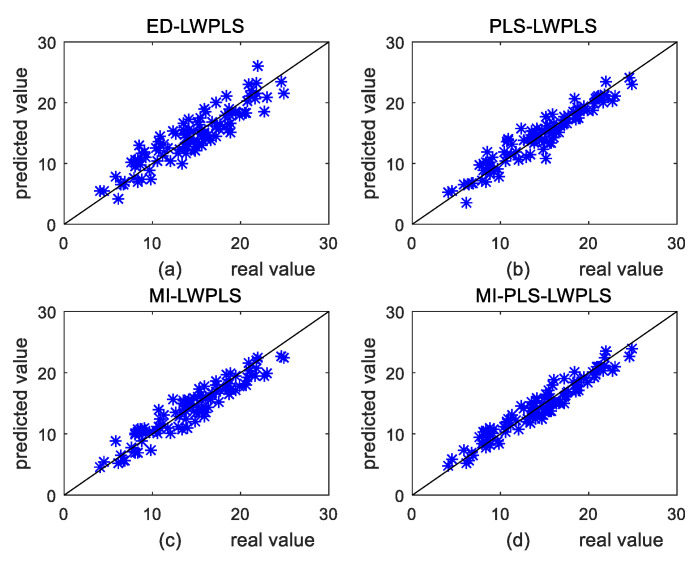
Comparison of prediction scatter plots in case 2: (**a**) Euclidean distance-based locally weighted partial least squares (ED-LWPLS); (**b**) PLS-LWPLS; (**c**) mutual information (MI)-LWPLS; (**d**) MI-PLS-LWPLS.

Figure 3 shows the scatter plots between real and predicted values of the four methods on the test set in case 2. Figure 3a shows the result of ED-LWPLS. It is observed that data points in Figure 3a are the most scattered among the four scatter plots, indicating that deviation between the predicted and real values is the largest. Prediction results of Figure 3b,c are close and both better than that of Figure 3a. Figure 3d shows the best prediction result since data points in Figure 3d are most concentrated near the diagonal line among the four plots. This also proves that the prediction accuracy of the MI-PLS-LWPLS method is the highest.

### 4.2. Industrial Case

#### 4.2.1. Debutanizer Column Process

A debutanizer is used in the process of desulfurization and naphtha separation. Butane concentration at the bottom of the tower is an important index to ensure the quality of process control, so it needs to be monitored in real-time [1,19]. However, traditional online measurement using meteorological chromatography is very time-consuming and does not meet the needs of real-time control.

**Figure 4:**
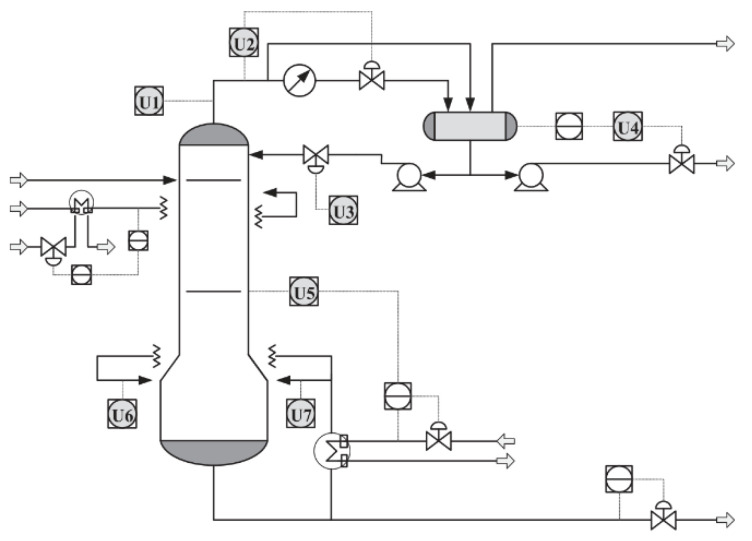
Debutanizer column process.

Therefore, a butane concentration measurement based on a soft sensor is an important alternative solution. To establish a soft sensing model for butane concentration measurement, seven variables that are easy to detect in the debutanizer were selected as auxiliary variables. The flow chart of the debutanizer column process (DCP) is shown in Figure 4, in which U1 ~ U7 are the installation locations of real-time monitoring devices for the seven auxiliary variables. An explanation of these seven variables is given in Table 2.

The DCP dataset, provided by [1], contains 2394 samples obtained from a DCP and has been a popular benchmark for evaluating various soft sensors [42,43,44,45]. The first half of samples was chosen as a training set, the remaining half was divided into two parts including a validation set for parameter optimization and a test set. The following model structure was adopted, in which the input variables were expanded according to the experience of experts [1,45].

(30) $$\overset{⌢}{y}(t)=f_{\mathrm{DCP}}\left(\begin{array}{c} U_{1}(t),U_{2}(t),U_{3}(t),U_{4}(t),U_{5}(t),U_{5}(t-1),U_{5}(t-2),\\ U_{5}(t-3),\frac{U_{6}(t)+U_{7}(t)}{2},y(t-4),y(t-5),y(t-6) \end{array}\right)$$

where *t* is the current sampling time, *y*(*t*) represents the actual butane concentration, *U_i_*(*t*) (*i* = 1, 2,..., 7) represents the sampling value of the *i*th input variable, and $\overset{⌢}{y}(t)$ is obtained by the soft sensing model, representing the predicted butane concentration.

**Figure 5:**
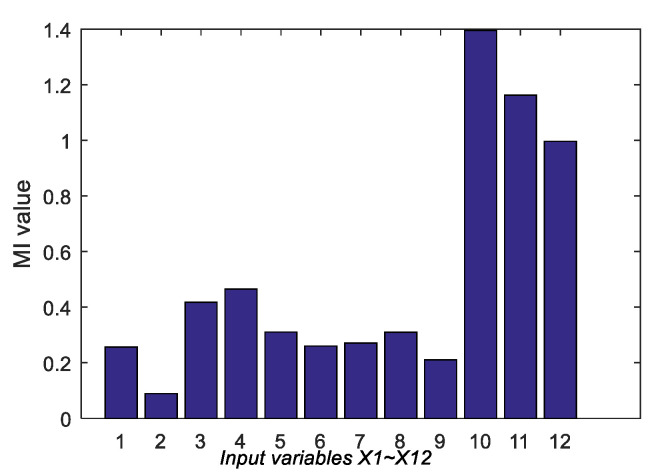
MI values between 12 input variables and y.

For ease of description, the 12 expanded input variables are noted as *X*_1_, *X*_2_,..., *X*_12_. Firstly, the correlation between *X_i_* (*i* = 1, 2,..., 7) and *y* was examined by calculating MI between them. Figure 5 shows the histogram of MI values between 12 input variables and *y*. One can see that MI values of different input variables vary greatly, indicating that they have different correlations with the output variable.

In this study, multicollinearity between input variables was also examined by using the common variance inflation factor (VIF) method. In this method, one of the input variables *X_i_* is taken as the output, then the other variables are used for regression to obtain an estimated value ${\overset{⌢}{X}}_{i}$, and then the variance inflation factor is calculated as follows:

(31) $${\mathrm{VIF}}_{i}=\frac{1}{1-R_{i}^{2}}$$

where $R_{i}^{2}$ is the determination coefficient obtained according to the regression result.

Generally, if there is at least one VIF*_i_* (*i* = 1, 2,…,12) greater than 10, it is considered that the input variables are multi-collinear. The VIF value of each input variable is shown in Table 3. One can see that there are several VIF values greater than 10, so multicollinearity does exist in DCP data.

**Figure 6:**
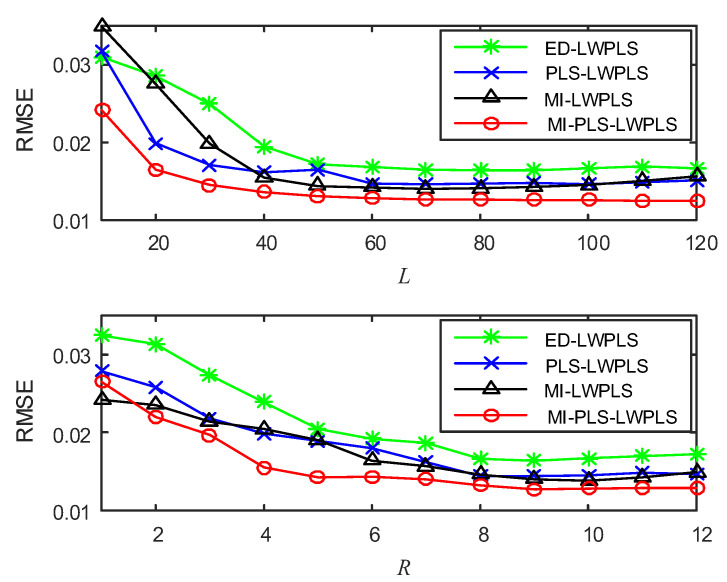
Changes of RMSEs with *L* and *R* in the validation set in the four methods.

To verify the effectiveness of MI-PLS-LWPLS, four modeling methods, ED-LWPLS, PLS-LWPLS, MI-LWPLS, and MI-PLS-LWPLS were used for soft sensor modeling on the DCP dataset, and their prediction results were compared. First of all, the values of parameters *L* (number of local modeling samples) and *R* (number of latent variables) in LWPLS needed to be determined. In this study, we selected the optimized parameter values by investigating RMSEs in the validation set. Figure 6 shows the change curves of RMSEs with different parameter values in the four methods. One can see that when the values of *L* and *R* are small, RMSEs of the four methods decrease with the increase of both *L* and *R* values. However, when *L* is greater than 60 or *R* is greater than 8, the changes of RMSEs are not significant. Therefore, the values of *L* and *R* in the four methods are determined as *L* = 60 and *R* = 8.

The tuning parameter *h* is determined by selecting an initial optimal value from the value set {0.01, 0.05, 0.1, 0.3, 0.6, 0.8, 1, 1.3, 1.6, 2, 5, 10, 20, 30, 50} to minimize the RMSE of the validation set, further reducing the selection range near the optimal value and finally determining the optimized h value. In this study, the optimal h values of the four methods ED-LWPLS, PLS-LWPLS, MI-LWPLS, and PLS-MI-LWPLS were finally selected as 0.05, 0.3, 0.01, and 2.6, respectively.

#### 4.2.2. DCP Experimental Results and Analysis

Table 4 gives statistical prediction errors of the four methods on both the validation dataset and test dataset. It is observed that the MI-PLS-LWPLS method achieves the minimum RMSE and MARE, indicating that its prediction result is the best. The RMSE and MARE of ED-LWPLS have the largest values among the four methods, indicating the worst prediction result. This is because the ED-LWPLS method only uses the input information of historical samples to calculate similarity by ED in the original variable space, and it does not consider the different correlations between input and output variables. PLS-LWPLS and MI-LWPLS both use the input and output information of historical samples to calculate the similarity, which greatly improves the prediction accuracy. However, the PLS-based similarity measure method ignores the nonlinear correlation between input and output, and the MI-based similarity measure method cannot deal with the collinear redundancy of input variables, so the prediction performance of these two methods needs to be further improved. The proposed MI-PLS-LWPLS method combines the advantages of PLS and MI in similarity calculation, so as to deal with collinearity of input variables and the nonlinear correlation between the input and output. Therefore, it has the best performance on the DCP dataset with multicollinearity.

**Figure 7:**
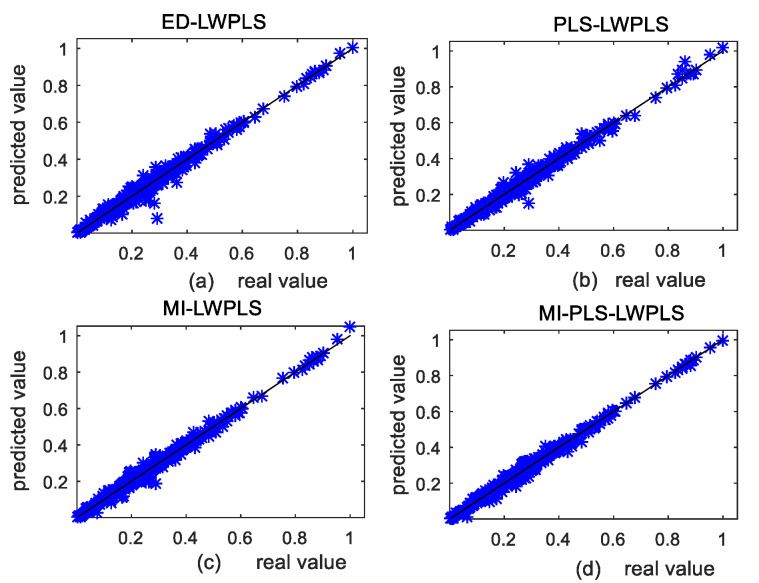
Comparison of prediction scatter plots for the test set of the four methods: (**a**) ED-LWPLS; (**b**) PLS-LWPLS; (**c**) MI-LWPLS; (**d**) MI-PLS-LWPLS.

Figure 7 shows scatter plots of prediction results for the test set of the four methods, (a) ED-LWPLS, (b) PLS-LWPLS, (c) MI-LWPLS, and (d) MI-PLS-LWPLS. By comparing the four scatter plots in Figure 7, one can see that the plot points in Figure 7d are most concentrated near the diagonal line, which proves that the MI-PLS-LWPLS method achieves the highest prediction accuracy. The performance of the other three methods needs to be improved on the complex DCP dataset with both nonlinearity and collinearity.

In order to investigate the computational efficiency of the proposed MI-PLS-LWPLS modeling method, we compared the predicted response time of the four methods. For each method, the time required to respond to the entire test set was recorded. Each method was run 20 times, and then the average value of the response time was taken, as shown in Table 5. It can be seen that compared with other LWPLS modeling methods based on different similarity measures, the prediction response time of the proposed method is only slightly increased. This is because the two-stage calculation strategy designed in this paper puts the operation related to historical samples in the training stage as much as possible, while the online calculation only aims at the transformation and operation closely related to query samples, which guarantees a fast response speed. Compared with the slow dynamic characteristics of the chemical process and low sampling frequency of quality parameters, the response speed can meet the requirements of process control.

## 5. Conclusions

This paper mainly focuses on the similarity measure in the JITL framework for soft sensor modeling. Firstly, several representative traditional similarity measure methods were analyzed. Through the analysis, it is known that in order to accurately calculate the similarity between samples, some key factors need to be considered comprehensively, including both consideration of input and output information, the different effects of input on output, the redundancy and collinearity of input variables, and the complexity of calculation. Based on the analysis of the shortcomings of current similarity measure methods, a new similarity measure method combining MI and PLS is proposed. The main contribution of the proposed method in solving the similarity measure problem is as follows:

(1) MI is used to calculate the correlation between input variables and the output, and the input variables are weighted by the MI value, so that the input and output information, as well as the different contribution of input to output, can be both considered in the similarity measure, and the uncorrelated redundant variables are eliminated.

(2) The weighted input variables are projected by the PLS algorithm, and the sample similarity is calculated in the latent variable space. This allows the influence of collinearity between input variables on the similarity measure to be eliminated. In the case of high-dimensional input, dimension reduction by PLS can also alleviate the complexity of calculation.

In order to use the above MI-PLS-based similarity measure method to develop a soft sensor under the JITL framework, we used LWPLS, which is commonly used in soft sensor modeling to build the local model. A two-stage modeling strategy was designed to reduce the online computing burden as much as possible, including a training stage and a prediction stage, so as to ensure a fast response to queries. In addition, in order to fully describe the relationship between the input and output, we adopted the following double weighting strategy, which considers the importance of both variables and samples.

(3) Weighted by MI, variables with high correlation with the output get larger weights, and weighted by similarity, samples more similar to the query have larger weights in building the local model. By giving larger weights to the more relevant variables and samples, the mapping relationship between process input and output can be better described, so the accuracy of the model is improved.

Finally, the effectiveness of the proposed method was verified by both numerical and industrial cases.

## Figures and Tables

**Table 1:** Prediction errors of the four methods.

Method	Case 1		Case 2	
	RMSE	MARE (%)	RMSE	MARE (%)
ED-LWPLS	1.98	13.28	2.01	13.58
PLS-LWPLS	1.61	11.13	1.55	10.15
MI-LWPLS	1.50	9.99	1.54	10.11
MI-PLS-LWPLS	**1.42**	**9.70**	**1.38**	**9.47**

**Table 2:** Auxiliary variables for the debutanizer column process.

Case 1	Case 2
U1	Top temperature
U2	Top pressure
U3	Reflux flow
U4	Flow to next process
U5	6th tray temperature
U6	Bottom temperature
U7	Bottom pressure

**Table 3:** Variance inflation factor (VIF) values of input variables.

**Input Variables**	*X*_1_	*X*_2_	*X*_3_	*X*_4_	*X*_5_	*X*_6_
**VIF**	1.6	1.2	1.5	1.3	**38.6**	**118.7**
**Input Variables**	*X*_7_	*X*_8_	*X*_9_	*X*_10_	*X*_11_	*X*_12_
**VIF**	**119.3**	36.4	3.4	**1078.5**	**3972.6**	**1020.7**

**Table 4:** Statistical analysis of prediction errors of the debutanizer column process (DCP) dataset.

Method	Validation Dataset		Test Dataset	
	RMSE	MARE (%)	RMSE	MARE (%)
ED-LWPLS	0.0164	5.81	0.0188	6.20
PLS-LWPLS	0.0146	5.27	0.0155	5.47
MI-LWPLS	0.0140	5.16	0.0153	5.42
MI-PLS-LWPLS	0.0129	4.10	0.0135	4.73

**Table 5:** Comparison of the response time of the four methods.

Method	Prediction Time (s)
ED-LWPLS	6.41
PLS-LWPLS	6.22
MI-LWPLS	7.19
MI-PLS-LWPLS	**7.32**
